# Supplementary material for: Red, Gold and Green: Microbial Contribution of Rhodophyta and Other Algae to Green Turtle (Chelonia mydas) Gut Microbiome
Source: Microorganisms. 2022 Oct 8;10(10):1988. doi: 10.3390/microorganisms10101988 (PMC9610419; doi:10.3390/microorganisms10101988)
Supplement: Supplementary file 1 [file microorganisms-10-01988-s001.zip › microorganisms-1895937-supplementary.pdf]

“Red, gold and green: microbial contribution of Rhodophyta and other algae to green turtle (*Chelonia mydas*) gut microbiome”

Supplementary material

Lucia Diaz-Abad[1,2], Natassia Bacco-Mannina[1], Fernando Miguel Madeira[3], Ester A. Serrão[1,4], Aissa Regalla[5], Ana R. Patrício[6,7], Pedro R. Frade[1,8*]

[1] CCMAR - Centre of Marine Sciences, CIMAR, University of Algarve, Faro, Portugal

[2] IMBRSea, International Master of Science in Marine Biological Resources

[3] cE3c - Centre for Ecology, Evolution and Environmental Changes & CHANGE - Global Change and Sustainability Institute, Faculdade de Ciências da Universidade de Lisboa, Lisbon, Portugal

[4] CIBIO/InBIO - Centro de Investigação em Biodiversidade e Recursos Genéticos, Universidade do Porto, Vairão, Portugal

[5] IBAP - Instituto da Biodiversidade e das Áreas Protegidas, Dr. Alfredo Simão da Silva, Bissau, Guinea-Bissau

[6] MARE - Marine and Environmental Sciences Centre, Ispa - Instituto Universitário, Lisbon, Portugal

[7] Centre for Ecology & Conservation, College of Life and Environmental Sciences, University of Exeter, UK.

[8] Natural History Museum Vienna, Vienna, Austria

* Correspondence: pedro.frade@nhm-wien.ac.at

Table S1. Summary information of the foraging green turtles sampled for this study. CCL – curved carapace length; CCW – curved carapace width.

Date of capture	Time of capture	Site of capture	Lat (N)	Long (W)	Animal code	Life-stage	PIT tag	CCL (cm)	CCW (cm)
27/10/2019	13:00 - 14:00	Ancante	11.3121	-16.4007	J1	Juvenile	977200009430768	39.4	37.6
27/10/2019	13:00 - 14:00	Ancante	11.3121	-16.4007	J2	Juvenile	977200009429926	36.5	32.5
27/10/2019	13:00 - 14:00	Ancante	11.3121	-16.4007	J3	Juvenile	977200009427885	38.3	34.3
27/10/2019	13:00 - 14:00	Ancante	11.3121	-16.4007	J4	Juvenile	977200009426947	40	35.9
27/10/2019	13:00 - 14:00	Ancante	11.3121	-16.4007	J5	Juvenile	977200009427231	48.1	41.5
27/10/2019	13:00 - 14:00	Ancante	11.3121	-16.4007	J6	Juvenile	977200009427097	39.5	35.7
27/10/2019	13:00 - 14:00	Ancante	11.3121	-16.4007	J7	Juvenile	977200009430232	40.6	36.2

Table S2. Summary table of samples collected from green turtles (in green) and from putative food items (in yellow) for microbiome assessment. Two different gastro-intestinal (GI) tract compartments were sampled from juvenile green turtles: cloaca and esophagus, while two other GI tract sections were sampled from dead hatchlings at the nesting beach: stomach and intestines. From one same animal, different sample types were collected, and the DNA extraction was performed individually for each sample type. For instance, there are two samples of the same animal J6, one for cloaca and one for esophagus, and for each one of them, a separated DNA extraction was performed. Following columns include the number of reads per sample before and after sequence filtering with DADA2 package in QIMME2. In column 'Sample code': **J** stands for juvenile and **H** stands for hatchling. Column 'Sampletype' represents the green turtle GI tract section from where each sample was collected and the "food item groups" into which the putative food items were pooled together for the analyses. 'Input' column corresponds to the initial number of reads in each sample; 'Denoised' column includes the number of sequences obtained after correcting sequencing errors; 'Merged' column includes the sequences that have aligned and combined fragments of a DNA sequences to reconstruct the original structure of the DNA and the and 'Non-chimeric' column corresponds to the final number of reads obtained after the sequencing filtering and the number of reads per sample used in the rest of the analyses.

**Sample code**	**Sampletype**	**Input**	**Filtered**	**Denoised**	**Merged**	**Non-chimeric**
J4	Esophagus	824022	260527	260352	225123	217347
J3	Cloaca	713666	91875	91835	79464	78136
J5	Cloaca	756856	98484	98478	93791	92433
H2	Stomach	769108	170386	170292	145100	140849
H2	Intestines	682181	207647	207022	172025	167071
J3	Esophagus	681401	196841	196757	179045	176725
J1	Esophagus	689904	211904	211747	189352	172745

J1	Cloaca	611032	92195	92185	76090	61359
J6	Cloaca	677126	115314	115311	94795	85661
J6	Esophagus	503366	8802	8802	2553	2553
J7	Esophagus	656563	194659	194530	166601	158237
J7	Cloaca	796908	218653	218202	180089	137582
J2	Esophagus	678394	170014	169900	149309	137343
J5	Esophagus	703085	197196	197078	178132	169713
J5	Cloaca	527620	153986	153581	134687	126650
H1	Stomach	605621	91022	90945	69351	69180
H3	Intestines	709503	88359	88359	82530	77447
H4	Stomach	703947	204399	204117	119735	116554
H5	Intestines	743018	34124	34124	9205	9205
J2	Cloaca	567334	135518	135264	102039	98149
Sargassum sp.	Brown	991746	322143	321857	266197	235865
Caulerpa sp.	Green	745138	229870	229191	199398	193607
Caulerpa sp.	Green	826524	343828	343401	342220	341949
Hypnea sp.	Red	470125	30817	30761	23293	19107
Caulerpa sp. 3	Green	791852	111426	111421	111037	111037
Caulerpa sp. 3	Green	692785	206227	205718	188160	184310
Halodule sp. 1	Seagrass	687592	163479	163451	145167	142466

Halodule sp. 1	Seagrass	506816	25188	25128	12254	8875
Halodule sp. 2	Seagrass	669378	90900	90869	79966	52142
Halodule sp. 2	Seagrass	743960	239930	239593	195881	185439
Ulva sp. 2	Green	693800	204215	203910	180894	176366
Padina sp. 5	Brown	736329	128965	128962	109144	79941
Colpomenia sp.	Brown	737636	240243	239301	169599	151734
Rhodophyta sp.	Red	670550	211600	211171	159270	143253
Dyctiota sp.	Brown	608223	197251	197154	169842	164084

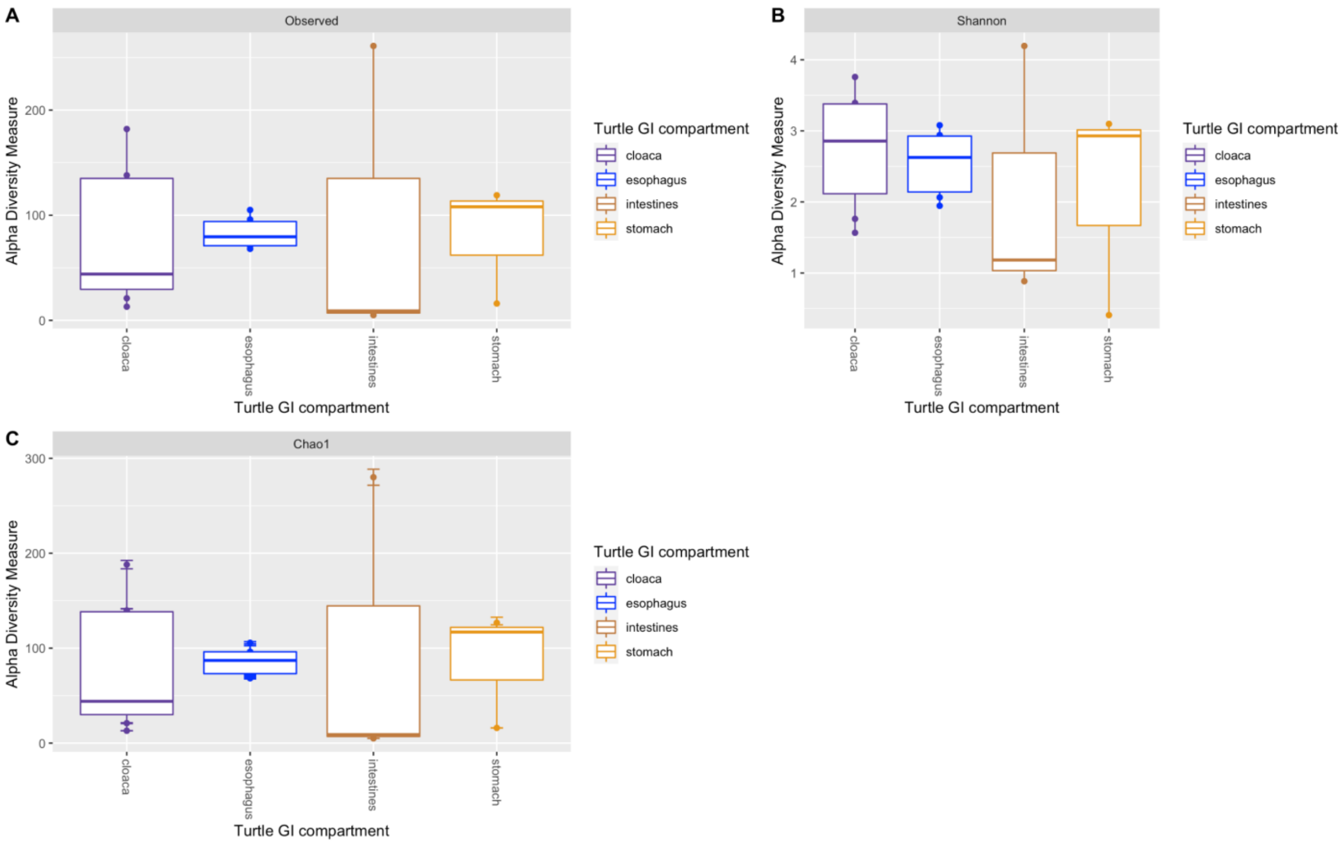

Figure S1. (**A)** Alpha-diversity boxplots for the four turtle GI compartments showing observed species richness (count of unique ASVs in each compartment). Although intestines samples exhibited higher richness than the other compartments and cloacal samples yielded the lowest diversity, overall, alpha diversity was similar among the GI turtle compartments. (**B)** Alpha-diversity boxplots for the four turtle GI compartments showing Shannon index. (**C)** Alpha-diversity boxplots for the four turtle GI compartments showing Chao 1 index.

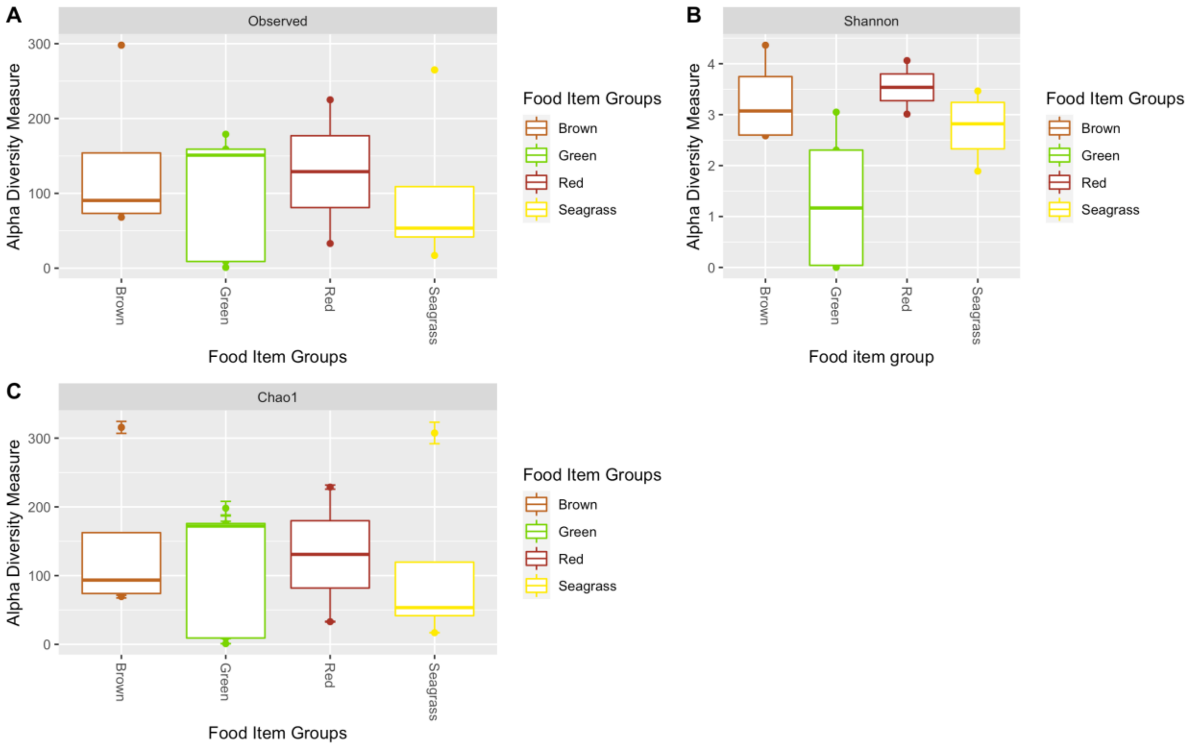

Figure S2. (**A)** Alpha-diversity boxplots for observed species richness (count of unique ASVs in each food item group) level. Although alpha diversity was not significantly different across the food groups, brown algae were the most diverse food item, and seagrass the lowest. (**B)** Alpha-diversity boxplots for the food items showing Shannon index. (**C)** Alpha-diversity boxplots for the food items showing Chao 1 index.

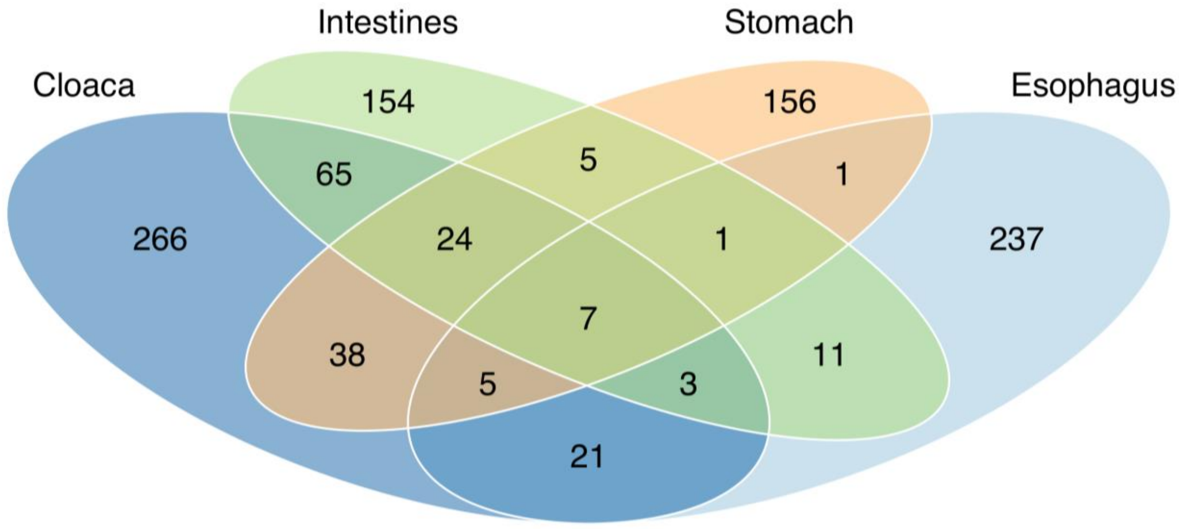

Figure S3. Sample type specific associated bacterial ASVs. Venn diagram exploring specific and ubiquitous ASVs between the four GI turtle compartments. Juvenile sections (esophagus and cloaca) presented higher content of unique ASVs than hatchling ones, and between all of them, only 7 ASVs were shared.

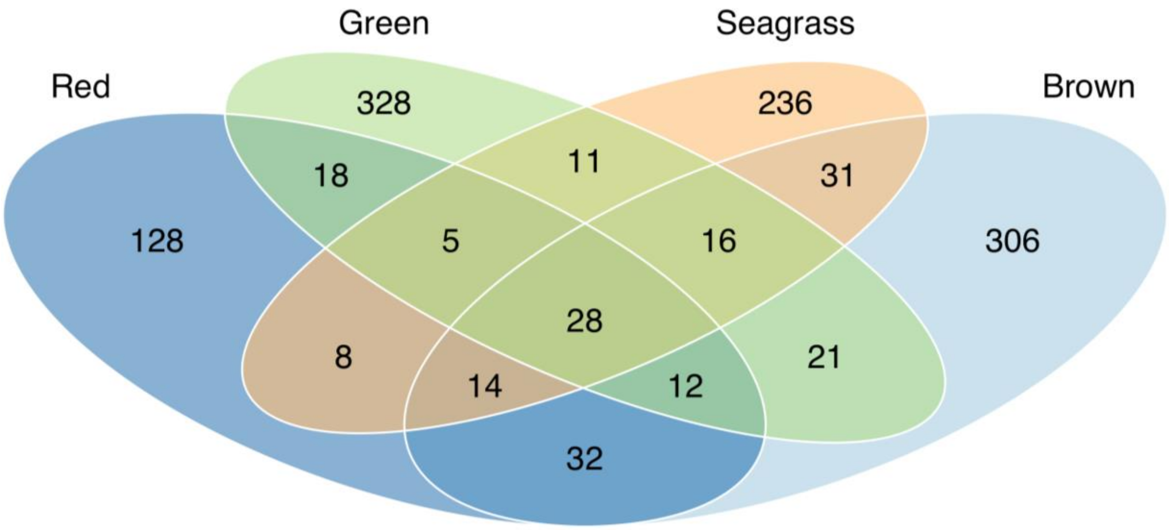

Figure S4. Sample type specific associated bacterial ASVs. Venn diagram exploring specific and ubiquitous ASVs between the four food item groups. Green algae showed the highest number of unique ASVs, while red algae presented the opposite. Across all food groups, there were 28 ubiquitous ASVs.

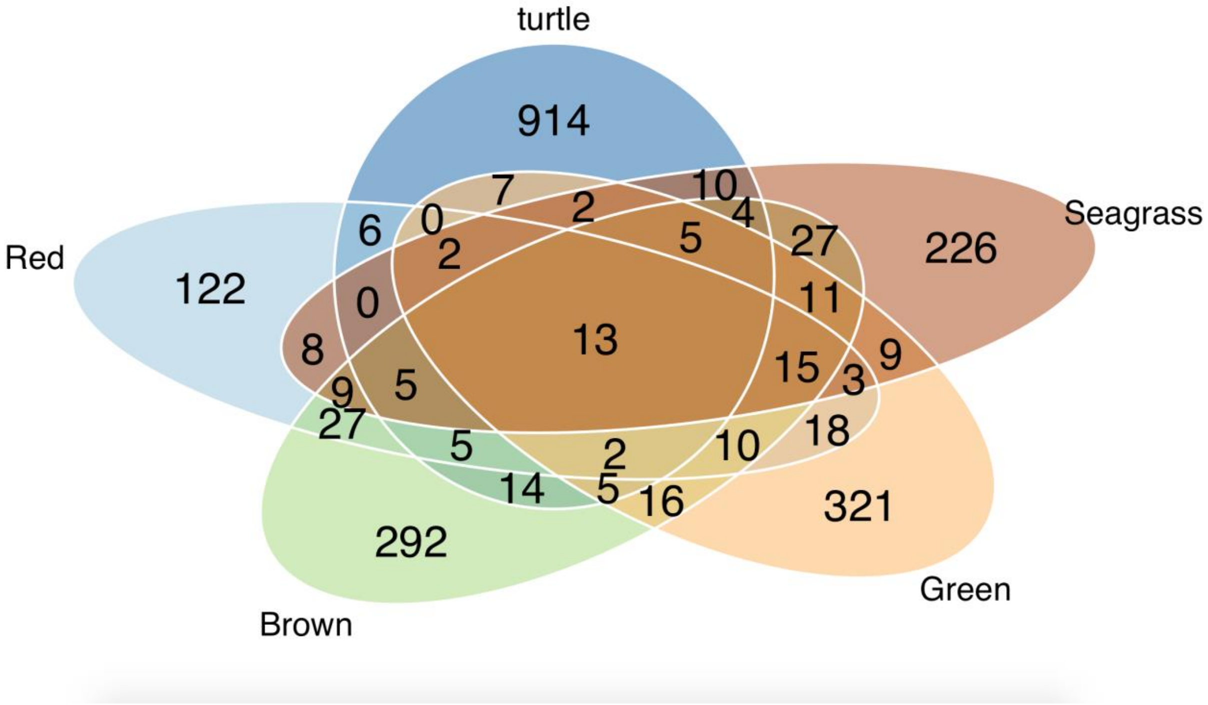

Figure S5. Sample type specific associated bacterial ASVs. Venn diagram exploring specific and ubiquitous ASVs between the four food item groups and the four turtle compartments together. 13 ASVs were shared across all sample types and 6 between red algae and all of the turtle compartments.

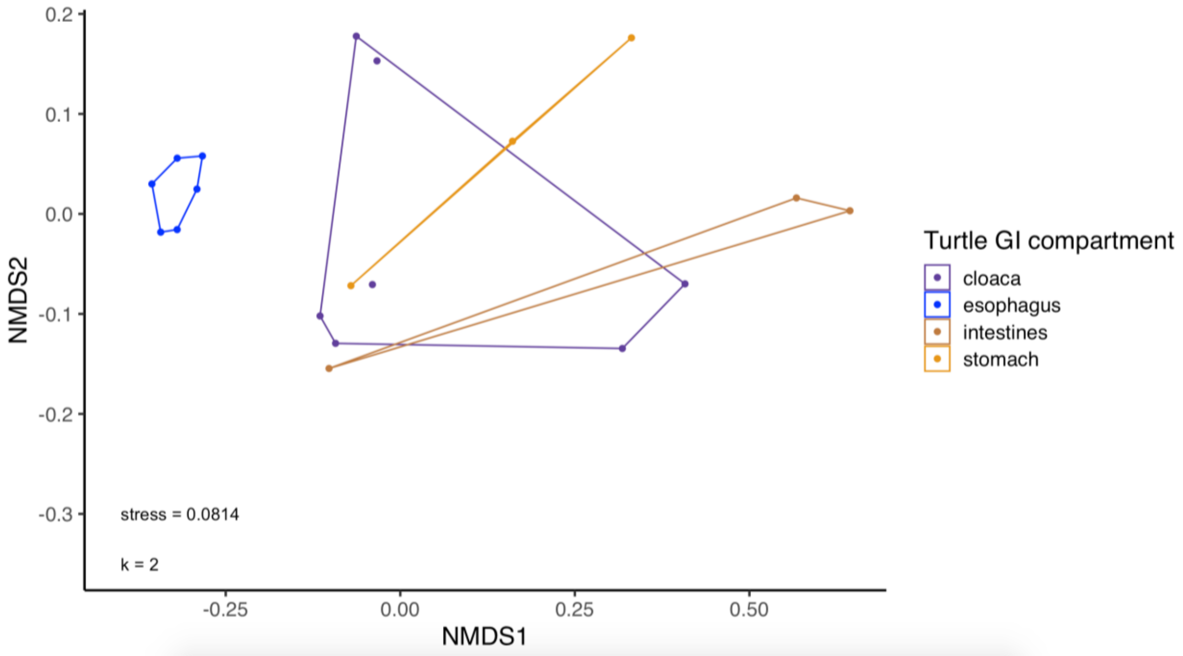

Figure S6. Microbial community structure of green turtles GI compartments: esophagus (n=6) cloaca (n=7), stomach (n=3) and intestines (n=3) samples. NMDS plot based on Bray-Curtis dissimilarity. Hatchlings (stomach and intestines) overlapped with cloaca, while esophagus presented its own homogeneous composition separated from the rest.

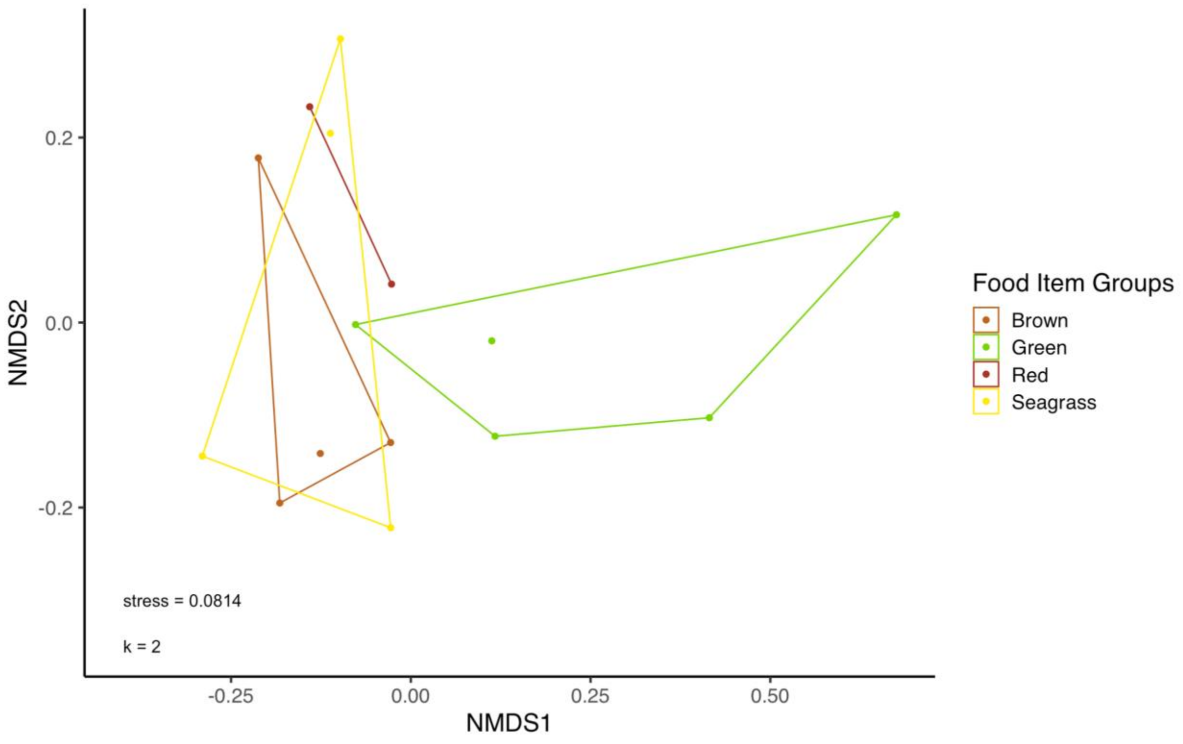

Figure S7. Microbial community structure of the green turtles food item groups: green algae (n=5), red algae (n=2), brown algae (n=4) and seagrass (n=4) samples. NMDS plot based on Bray-Curtis dissimilarity. Both brown and red algae overlapped with seagrass separately, while green algae were separated from the other food groups.

Table S3. PERMANOVA pairwise comparisons (after Bonferroni correction) across all of the different sample types (turtle GI compartments and food item groups). Significant differences in the microbial community structure only happened between juvenile samples and green algae, while the rest of the GI compartments and food item groups had no significant differences in their microbial community compositions.

	Red alage	Green alage	Brown alage	Seagrass
**Esophagus**	**F(1,7)=5.24, p=0.61**	**F(1,9)=4.28, p=0.04**	**F(1,8)=4.47, p=0.08**	**F(1,8)=4.12, p=0.07**
**Cloaca**	**F(1,7)=1.75, p=0.06**	**F(1,10)=1.66, p=0.04**	**F(1,9)=1.65, p=0.19**	**F(1,9)=1.55, p=0.26**
**Stomach**	**F(1,3)=1.55, p=0.10**	**F(1,6)=1.25, p=0.16**	**F(1,5)=1.70, p=0.23**	**F(1,5)=1.20, p=0.20**
**Intestines**	**F(1,3)=1.67, p=0.10**	**F(1,6)=1.35, p=0.15**	**F(1,5)=1.42, p=0.09**	**F(1,5)=1.31, p=0.09**

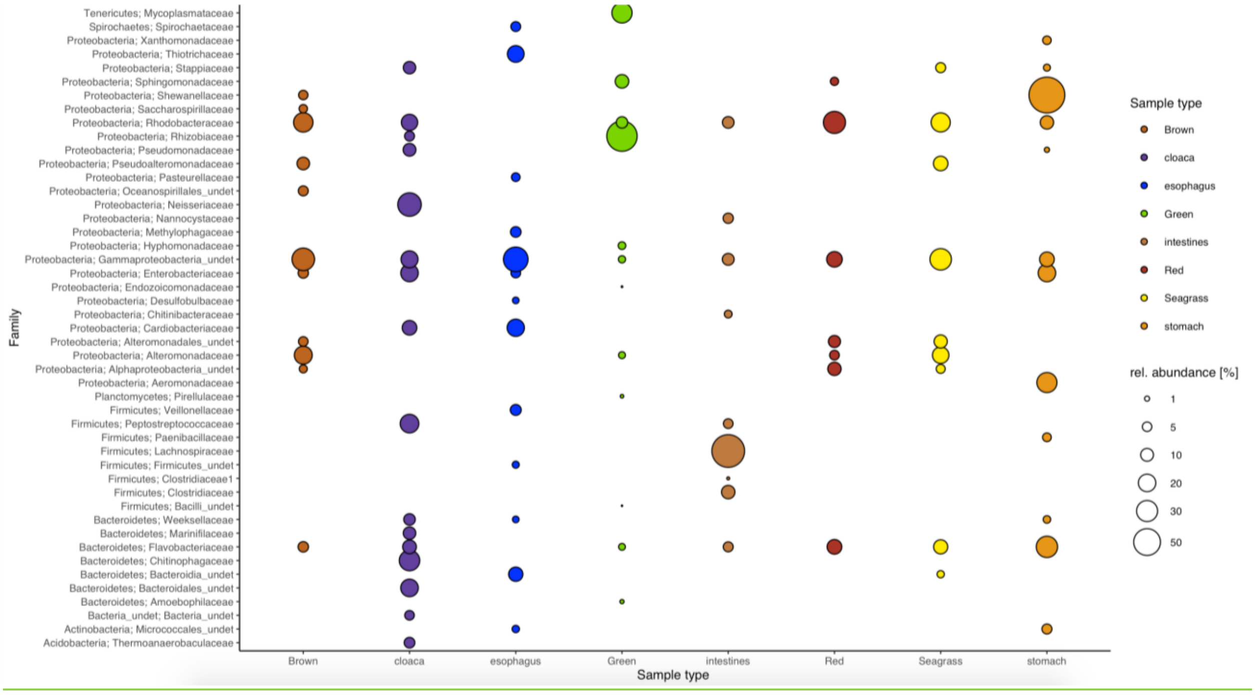

Figure S8. Microbial community composition at the family level (Y axis, Phylum;Family) across all of the sample types of this study. Average of the abundance of the families across the samples of each sample type is represented as relative abundance. Abundance values <1% are not represented. Proteobacteria and Bacteroidetes were the two bacterial phyla present across all of the sample types, which happened to be the most abundant ones. Only one undetermined family was present across all sample types belonging to Proteobacteria phylum: Gammaproteobacteria_undet.

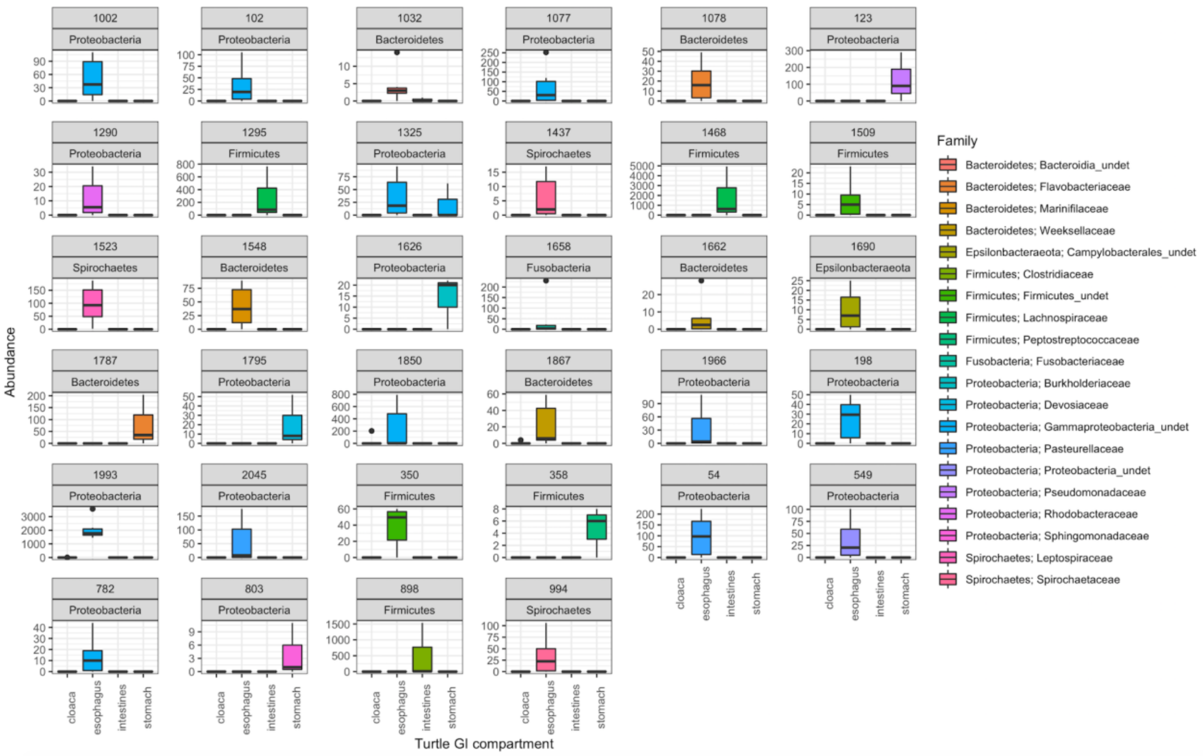

Figure S9. Indicator bacterial ASVs for the turtle GI compartments. Number of indicator and its Phyla appear at the top of each box plot. The corresponding family of each indicator is listed in the legend. Each boxplot corresponds to one indicator and the compartments it appears on, labelled in the X axis. Abundance of the indicator in the expressed turtle compartment is shown in the Y axis. 34 indicator ASVs were detected in total, being distributed throughout the GI compartments as following: 24 in esophagus, 6 in stomach, 3 in the intestines and 1 shared between the stomach and esophagus.

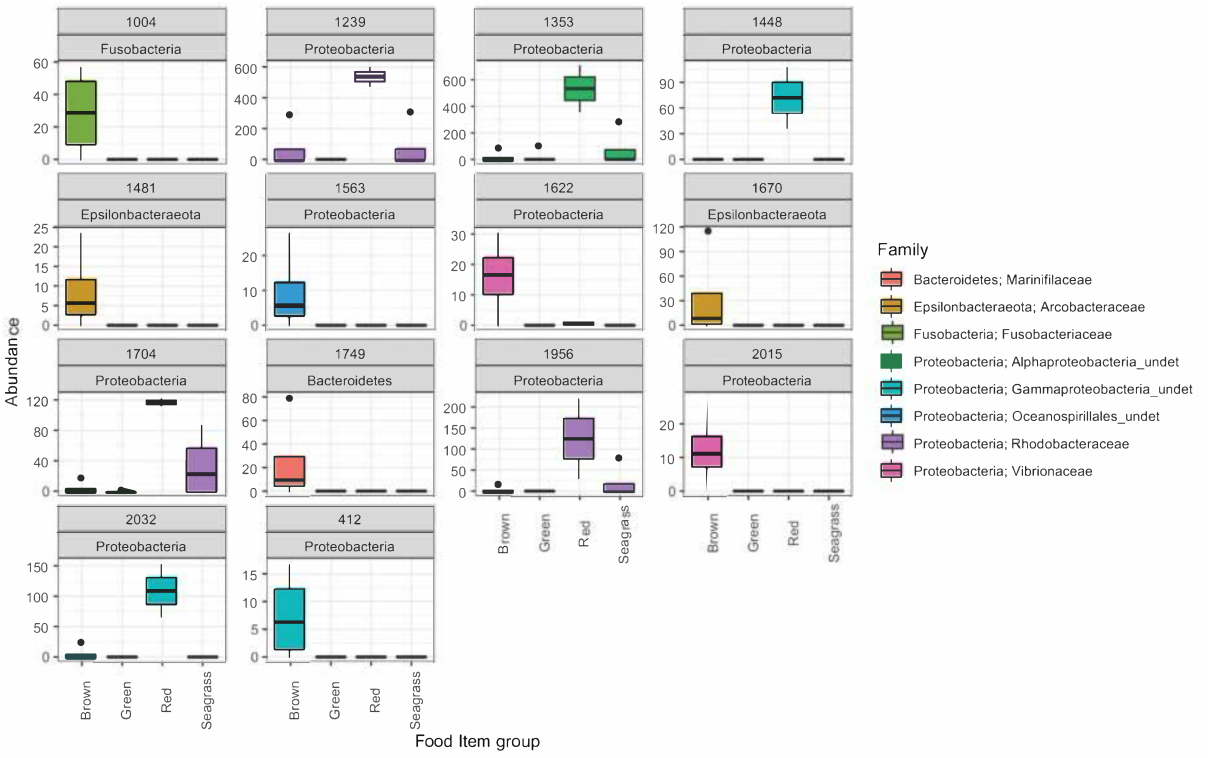

Figure S10. Indicator bacterial ASVs for the food item groups. Number of indicator and its Phyla appear at the top of each box plot. The corresponding family of each indicator is listed in the legend. Each boxplot corresponds to one indicator and the compartments it appears on, labelled in the X axis. Abundance of the indicator in the expressed food item group is shown in the Y axis. In total, 14 indicator ASVs were observed for the food item groups, 8 corresponding to brown algae and 6 to red.

DESeq2 Analysis

Table S4. Differential Abundance in ASVs expression analysis (DESeq2) summary results. Most abundant significant bacterial families for each sample type causing microbial community differences between the different sample types are shown.

Sample type	Significant abundant bacterial phyla and families causing microbial community differences between sample types
**Esophagus**	Cardiobacteriaceae, Thiotrichaceae
**Cloaca**	Nannocystaceae
**Intestines**	Lachnospiraceea
**Stomach**	Enterobacteriaceae, Peptostreptococcaceae
**Red algae**	Gammaproteobacteria_undet, Flavobacteriaceae
**Brown algae**	Planctomycetes, Acidobacteria, Verrucomicrobia, Saccharospirillaceae
**Green algae**	Planctomycetes
**Seagrass**	Planctomycetes, Cyanobacteria

Microbial baselines

Table S5. Microbial baselines for juvenile samples of green turtle. Results for community composition (two most abundant families, mean relative abundance in %), indicator species analysis (INDVAL; family/species level) and differential abundance in ASVs expression analysis (DESeq2; family level) are shown.

	**Esophagus**	**Cloaca**
**Abundant families**	Gammaproteobacteria_undet (16.1±6.9%), Cardiobacteriaceae (6.2±17%)	Gammaproteobacteria_undet (6±11.2%), Rhodobacteraceae (5.5±10.8%)
**INDVAL**	Weeksellaceae_unde, Gammaproteobacteria_undet, Shewanella_undet, Flavobacteriaceae_undet, Rhodobacteraceae_undet, Flavobacteriaceae_undet, Corynebacterium1_undet, Saccharimonadaceae_undet, Alphaproteobacteria_undet, Bdellovibrio_undet, Maritimimonas_undet, Alphaproteobacteria_undet, Nannocystaceae_undet, Octadecabactersp.UDC459, Unculturedalphaproteobacterium, Desulfatiferula_berrensis, Acinetobacter_undet, Acidobacteria_undet, Muricauda_undet, Hyphomicrobiaceae_undet, Proteobacteria_undet	
**DESeq2**	Cardiobacteriacea and Thiotrichaceae	Nannocystaceae

Table S6. Microbial baselines for hatchling samples of green turtle. Results for community composition (two most abundant families, mean relative abundance in %), indicator species analysis (INDVAL family/species level) and differential abundance in ASVs expression analysis (DESeq2; family level) are shown.

	**Stomach**	**Intestines**
**Abundant families**	Shewanellaceae (5.9%), Gammaproteobacteria_undet (2.6±14%)	Lachnospiraceae (9.77±19.4%), Gammaproteobacteria_undet (1.6±7.4%)
**INDVAL**	Arcobactersp.ONE, Bacteria_undet, Pseudoalteromonas_undet, Bacteria_undet, Flavobacteriaceae_undet, Bacteriovoracaceae_undet	Sandaracinaceae_undet, Sphingobacterium_undet, Acidobacteria_undet
**DESeq2**	Enterobacteriaceae, Peptostreptococcaceae	Lachnospiraceae

Table S7. Microbial baselines for food items. Results for community composition (two most abundant families, mean relative abundance in %), indicator species analysis (INDVAL; family/species level) and differential abundance in ASVs expression analysis (DESeq2; phylum/family level) are shown.

	**Red algae**	**Brown algae**	**Green algae**	**Seagrass**
**Abundant families**	Rhodobacteraceae (5.4±10.4%), Gammaprotecobacteria_undet (2.5±8.2%)	Gammaprotecobacteria_undet (11.2±15%), Rhodobacteraceae (4.1±1.5%), exclusive: Shewanellaceae (0.39%), Saccharospirillaceae (0.30%), Oceanospirillales_undet families (0.46%)	Exclusive and abundant: Rhizobiaceae family (21±45.5%), Mycoplasmataceae (6.64%) Exclusive: Amoebophilaceae, Hyphomonadaceae and Pirellulaceae (<10%)	Gammaprotecobacteria_undet (7.5±20.3%), Rhodobacteraceae (5.9±20%), exclusive: Stappiaceae family (0.46%)
**INDVAL**	Rhodobacteraceae_undet, Alphaproteobacteria_undet, Gammaproteobacteria_undet, Rhodobacteraceae_undet, Rhodobacteraceae_undet, Gammaproteobacteria_undet	Gammaproteobacteria_undet, Propionigenium_undet, Rhodobacteraceae_undet, Oceanospirillales_undet, vibrio_undet, Arcobacter_undet, Marinifilumsp.JNU_J034, vibrio_undet		
**DESeq2**	Gammaproteobacteria_undet and Flavobacteriaceae	Planctomycetes, Acidobacteria, Verrucomicrobia and Saccharospirillaceae	Planctomycetes	Planctomycetes, Cyanobacteria
